# Accuracy of Radiomics-Based Machine Learning for Predicting Risk of Recurrence in Non–Small Cell Lung Cancer: Systematic Review and Meta-Analysis

**DOI:** 10.2196/84223

**Published:** 2026-04-02

**Authors:** Junpei Wu, Ye Zhang, Jiaye Wang, Chengshui Chen, Shiyu Hu, Wenyu Chen

**Affiliations:** 1 Department of Pulmonary and Critical Care Medicine, Yiwu Central Hospital, Yiwu, China; 2 Department of Respiratory, The Affiliated Hospital of Jiaxing University, 1882 Zhonghuan South Road, Nanhu District, Jiaxing, China, 86 18358323756; 3 Health Management Center, The Affiliated Hospital of Jiaxing University, Jiaxing, China; 4 Department of Internal Medicine, Zhejiang Chinese Medical University, Hangzhou, China; 5 Department of Respiratory, The First Affiliated Hospital of Wenzhou Medical University, Wenzhou, China

**Keywords:** non–small cell lung cancer, NSCLC, machine learning, ML, radiomics, recurrence, prognostic prediction

## Abstract

**Background:**

During the diagnosis and treatment of non–small cell lung cancer (NSCLC), detecting the risk of its recurrence in an early phase is still challenging. Recent studies have investigated the radiomics-based machine learning (ML) models for detecting the risk of recurrence in NSCLC. However, there is still insufficient systematic evidence to prove its efficiency.

**Objective:**

This study is designed to systematically evaluate the effectiveness of radiomics-based ML in predicting the risk of recurrence in NSCLC, aiming to provide evidence-based support for the subsequent development of scoring tools to forecast recurrence risk.

**Methods:**

For acquiring research on radiomics-based models for forecasting the risk of recurrence in NSCLC, Cochrane Library, Web of Science, PubMed, and Embase were systematically retrieved, up to October 24, 2025. Studies on analyzing the recurrence of NSCLC using radiomics-based ML were included, while those in which only texture analysis was conducted or radiomics-based ML was not constructed were excluded. The Radiomics Quality Score (RQS) was used to appraise the eligible studies. Subgroup analyses were conducted according to the variables of the model, the background of treatment, the stage of lung cancer, and the pathological type.

**Results:**

Ultimately, 30 eligible studies in total were included, covering 7964 patients with NSCLC. According to the meta-analysis, the c-index of radiomics-based ML models for forecasting the risk of recurrence in NSCLC was 0.850 (95% CI 0.834‐0.866, 95% prediction interval [PI] 0.623‐1.004) in the training set. Specifically, the pooled c-index was 0.876 (95% CI 0.853‐0.900) among the patients receiving the stereotactic body radiation therapy and 0.825 (95% CI 0.804‐0.848) among those who received surgeries combined with other adjuvant treatment regimens. The c-index of the radiomics-based ML models combined with clinical features for forecasting the risk of recurrence in NSCLC was 0.833 (95% CI 0.822‐0.854, 95% PI 0.717‐0.945) in the training set. In contrast, the c-index of radiomics-based ML models for forecasting the risk of recurrence in NSCLC was 0.878 (95% CI 0.854‐0.902, 95% PI 0.681‐1.000) in the validation set. The c-index of radiomics-based ML models combined with clinical features for forecasting the risk of recurrence in NSCLC was 0.854 (95% CI 0.830‐0.878, 95% PI 0.655‐0.992) in the validation set. The average RQS across the included studies was 27.4%, revealing methodological limitations and an absence of standardization.

**Conclusions:**

This study is the first to confirm that radiomics-based ML models effectively predict the risk of recurrence in NSCLC. This study provides evidence-based support for the subsequent development or updating of radiomics-based ML models. However, the current methodological application of radiomics remains concerning. Therefore, in the future, research should standardize the workflow for implementing radiomics-based ML and incorporate multicenter imaging data to enhance its generalizability.

## Introduction

As documented in the Cancer Statistics Report 2024, lung cancer serves as a major contributor to global cancer-related mortality and ranks second in the incidence of new cancer cases among male individuals [1]. With an 85% incidence among individuals with lung cancer, non–small cell lung cancer (NSCLC) emerges as the most frequently observed pathological subtype, and its incidence is gradually increasing [2]. Hence, enhancing the prevention, screening, and management of NSCLC is clinically significant.

The current treatment methods for NSCLC are constantly evolving. Early-stage NSCLC surgeries and radiotherapy, which are traditional treatment approaches, are still widely used and have helped numerous patients achieve promising therapeutic outcomes. In recent years, emerging therapies such as molecular targeted therapy, antitumor angiogenesis therapy, and immunotherapy [3-5] for NSCLC have significantly progressed. Those innovative treatment methods offer patients more options and significantly improve their survival rates and quality of life. Although those treatment methods have led to significant survival benefits for patients with NSCLC, there are still some serious adverse prognoses, posing significant challenges in the formulation of treatment plans in clinical practice [6,7]. In particular, recurrence is a serious adverse outcome in NSCLC treatment. According to the epidemiological statistics on lung cancer, the 5-year cumulative recurrence rate of NSCLC (stage I) after surgery is 25% to 30%, and it is as high as 60% to 70% for NSCLC (stage IIIA) [8,9]. Therefore, early identification of the risk of recurrence in NSCLC is clinically significant for formulating individualized therapeutic schedules as well as rehabilitation plans.

Unfortunately, up to now, there has been a lack of tools for effectively predicting the recurrence of NSCLC in clinical practice. In recent years, with artificial intelligence evolving, radiomics has received widespread attention in clinical practice. Radiomics refers to the process of converting a group of high-data-content medical images into digital ones and combining machine learning (ML) to aid in the diagnosis of diseases, prediction of progression, and prognosis of diseases [10]. Radiomics was originally proposed by Lambin et al [11] from the Netherlands in 2012. In comparison with traditional models, radiomics-based models are capable of extracting imaging features through high-throughput methods, which can more comprehensively reflect the spatial heterogeneity of tumors and are equipped with higher information dimensions and discriminative capabilities. Therefore, it has shown significant advantages in multiple studies. The study by Chen et al [12] has revealed a high c-index of radiomics-based ML models for forecasting the risk of recurrence in NSCLC (the stage of early epidermal growth factor receptor mutation). In the study by Yan et al [13], a radiomics-based ML is effective for predicting lymph node metastasis in NSCLC.

Beyond conventional ML algorithms based on radiomics, recent research has also explored more advanced deep learning techniques and AI algorithms capable of perceiving uncertainty for medical prediction. These models use dimensionality and techniques of size reduction for feature selection, maintaining robustness even in datasets with incomplete or noisy information. For instance, deep ML algorithms integrating feature elimination and dimensionality reduction have been successfully applied to the diagnosis of thyroid cancer with randomly missing data. Additionally, big data-driven supervised ML models based on similar mechanisms have demonstrated high diagnostic performance in nonalcoholic steatohepatitis [14,15]. These advances highlight the potential of incorporating high-dimensional feature learning and uncertainty modeling into frameworks of medical prediction. However, their application in forecasting the recurrence of NSCLC remains limited, and no systematic review has been conducted to synthesize current evidence.

Against this backdrop, some researchers have sought to establish radiomics-based ML models to forecast the risk of recurrence in NSCLC. However, current studies are restricted by limited sample size, heterogeneous design, and data sourced from a single center [16]. These studies are absent from systematic quantitative integration and heterogeneity analysis, thereby failing to provide high-quality and evidence-based support. This poses a challenge to the development of intelligent models for predicting the risk of recurrence in NSCLC. Therefore, this study is designed to systematically evaluate the predictive performance of radiomics-based ML models for the recurrence risk of NSCLC and their current application in this field. This study provides evidence-based support for the future standardized application of radiomics and the development or update of prediction and assessment tools based on radiomics-based ML.

## Methods

### Registration

This study has been prospectively registered on the International Prospective Register of Systematic Reviews (PROSPERO) and was implemented strictly following the PRISMA (Preferred Reporting Items for Systematic Reviews and Meta-Analyses) 2020 guidelines to guarantee transparency and repeatability (Checklist 1) [17].

### Eligibility Criteria

#### Inclusion Criteria

The inclusion criteria were as follows: (1) individuals pathologically or clinically diagnosed with NSCLC; (2) studies on the recurrence in NSCLC using radiomics-based ML; (3) studies reporting outcome indicators of the accuracy of ML; (4) cohort studies, case-control studies, or cross-sectional studies; and (5) studies reported in English.

#### Exclusion Criteria

The exclusion criteria were as follows: (1) unpublished conference abstracts were included; (2) in studies, an ML model only based on clinical features was constructed for predicting the risk of recurrence in NSCLC, and a radiomics-based ML model was not established; (3) studies failed to strictly distinguish NSCLC from other types of lung cancers; and (4) in studies, outcome indicators were absent from appraising the predictive precision of an ML model, including c-index, sensitivity, specificity, accuracy, recovery rate, precision rate, confusion matrix, and *F*_1_-score.

### Data Sources and Search Strategy

To ensure transparency, the literature search was conducted in accordance with the PRISMA-S (Preferred Reporting Items for Systematic Reviews and Meta-Analyses–Search Extension) guidelines (Checklist 2) [18]. PubMed, Cochrane Library, Embase, and Web of Science were systematically searched for studies on predicting NSCLC recurrence based on radiomics from the inception of the databases to October 24, 2025, with no restrictions on region or year of publication. No specialized study registries were searched, nor were relevant experts contacted to obtain unpublished data. The reference lists of all included studies were manually checked (backward searching) to avoid missing potentially relevant studies. The search strategy was a combination of subject terms with relevant free terms. The subject terms comprised lung neoplasms (MeSH), machine learning (MeSH), and recurrence (MeSH). The search strategy for each database and the retrieval process are detailed in Table S2 in Multimedia Appendix 1 and Checklist 2. Prepublished search filters or search strategies for other literature reviews were employed, irrespective of language or study design.

### Literature Screening and Data Collection

The retrieved studies were imported into EndNote (Clarivate). After the elimination of duplicates based on title, author, and publication year, the studies were systematically reviewed for their titles and abstracts. Ultimately, the eligible studies were determined through a full-text review. In the included studies, recurrence was defined as recurrence of tumors within or adjacent to the treatment zone, mediastinal recurrence, or distant metastasis following curative-intent treatment such as surgery, chemoradiotherapy, or stereotactic body radiotherapy. Recurrence was generally identified according to the criteria of response evaluation criteria in solid tumors or confirmed via histopathology. To ensure accuracy, in several studies, double-blind evaluation was performed independently by 2 radiologists or oncologists, with disagreements resolved through negotiation. Only studies reporting a clear definition of recurrence and an explicit criterion for follow-up were included in this meta-analysis. To address the high dimensionality of radiomics data, procedures of multistep feature selection and dimensionality reduction were used across the included studies. Commonly applied methods included reproducibility filtering via intraclass correlation coefficient, redundancy removal based on correlation, univariate statistical screening, and feature selection based on ML, such as least absolute shrinkage and selection operator, recursive feature elimination, or principal component analysis. These approaches reduced feature space, enhanced the steadiness of models, thereby ensuring that only the most robust and clinically relevant features were utilized in the construction of prediction models.

Before extracting the data, a standardized spreadsheet for data extraction was created, including title, DOI, first author, country of first author, publication year, study design, follow-up duration, source of radiomics, number of participants involved in image segmentation, image segmentation software, case number of NSCLC in the training set, case number of NSCLC in the validation set, method of generating the validation set, presence or absence of overfitting, total number of participants in the validation set, method of variable selection, method of model construction, presence or absence of a radiomics score, presence or absence of other features combined for model construction (such as clinical features and genetic features), presence or absence of assessment of clinical applicability, presence or absence of evaluation of model calibration, presence or absence of overfitting assessment of the model, c-index, sensitivity, specificity, and diagnostic contingency table.

Literature screening and data extraction were implemented independently by two investigators (J Wu and SH), followed by a cross-verification to ensure consistency. All discrepancies were resolved by a third researcher (YZ) to reach a consensus.

### Evaluation for Study Quality

Two investigators used the Radiomics Quality Score (RQS) to assess the methodological quality and analyze risk of bias among the included studies, followed by a cross-verification. Any discrepancy was resolved by a third researcher to make a final decision. The RQS covered 16 items and described the entire process of radiomics, with a maximum score of 36 points.

### Synthesis Methods

Meta-analysis was performed for the c-index, an index for appraising the overall precision of ML models. In some original studies, when a 95% CI and a SE of the c-index were not reported, the SEs were estimated based on the study by Debray et al [19]. Among the ML models, discrepancies were identified in the selected variables, with parameters varying. Therefore, in terms of meta-analysis of the c-index, this study privileged the random effects model (REM). Furthermore, the results were calibrated using the Hartung-Knapp-Sidik-Jonkman approach, and the prediction intervals were presented. Subsequently, the bivariate mixed effects model was additionally used to conduct a meta-analysis for sensitivity and specificity. The meta-analysis was carried out via Stata 15.0 (StataCorp LLC).

## Results

### Literature Selection

**Figure 1.**
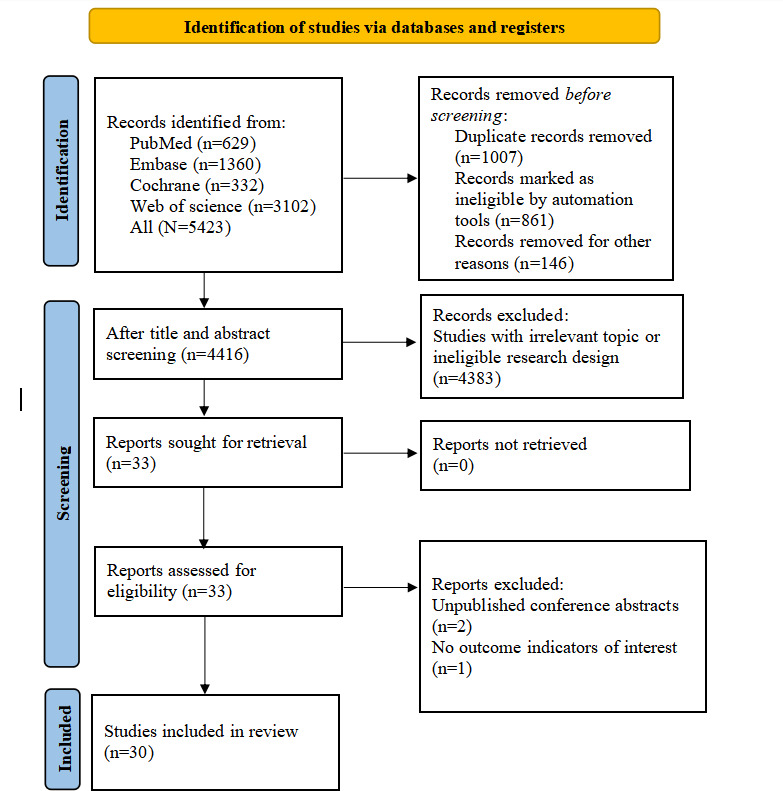
PRISMA (Preferred Reporting Items for Systematic Reviews and Meta-Analyses) flowchart for literature selection.

A total of 5423 reports were retrieved, among which 1007 duplicate studies were removed, with 4416 studies remaining. A total of 4383 studies were screened out according to the titles and abstracts, due to irrelevance to the study topic or an unmatched study design. The full texts of the remaining 33 studies that were preliminarily in line with the inclusion criteria were obtained. Through the review of the full texts, 2 unpublished conference abstracts and 1 study without outcome indicators were excluded. Ultimately, 30 studies were included. The process of literature selection is depicted in Figure 1.

### Features of the Included Studies

Those 30 selected studies were all published between 2019 and 2025, in 9 countries. The study types were all cohort studies. The study data were from multiple centers in 11 studies, from a single center in 17 studies, and from a registration database in 2 studies. A total of 8 studies clearly delineated the pathological types of lung adenocarcinoma and/or lung squamous cell carcinoma, while 22 did not delineate the pathological type of the tumor, merely describing it as NSCLC. A total of 19 studies included early-stage lung cancer (based on tumor node metastasis [TNM] staging in stages I-II), 9 included locally advanced or advanced lung cancer (based on TNM staging in stages III-IV), and 2 did not specify the tumor stage. Among the included studies, 16 discussed tumor recurrence after the stereotactic body radiation therapy (SBRT), and 22 discussed tumor recurrence after surgery and/or radiotherapy and chemotherapy. The follow-up time was mainly concentrated between 2 and 70 months. The image sources of the 26 studies mainly included computed tomography (CT) and positron emission tomography–computed tomography (PET-CT). Among them, 24 studies used CT, 2 studies used PET-CT, and 4 studies used both CT and PET-CT. The generation method of the validation set mainly involved random sampling and cross-validation. Among them, 6 studies included external validation (Table S2 in Multimedia Appendix 1).

### Evaluation of the Quality of the Selected Studies

None of the included studies described the use of multiple segmentation approaches, repeated measurements at different time points, or pretests with varying image parameters. None of them conducted prospective registration, provided a description of the degree of consistency with the “gold standard” method, or conducted cost-effectiveness analysis. Therefore, none of the included studies received any points in these categories. In 7 studies, only 1 doctor was involved in the image segmentation, and thus, they did not receive any points. Only the study by Aonpong et al [20] discussed the correlation between radiomics and tumor biology. A total of 16 studies did not report the radiomics score and therefore received a score of 0 in this category. All the included literature reported the discriminant statistics and their statistical significance, but only 15 studies applied the multiple sampling method. Accordingly, in this domain, 15 studies were awarded 2 points, while the remaining studies received 1 point. A total of 5 studies reported calibration statistics and were awarded 2 points, whereas the remaining studies received no points. Among the 30 included studies, all verified the radiomics features, with 1 study using 3 external datasets for verification, another study using 2 external datasets, and 7 studies using 1 external dataset for verification. A total of 7 studies performed decision curve analysis to evaluate the potential clinical utility of the models, each receiving 2 points. A total of 24 studies disclosed images and segmentation, earning 2 points each. One study disclosed data codes, receiving 3 points. A total of 5 studies did not disclose codes, data, or segmentation methods and therefore received no points. The final average score was 27.5% (Table S3 in Multimedia Appendix 1).

### Meta-Analysis: Radiomics-Based ML

**Figure 2.**
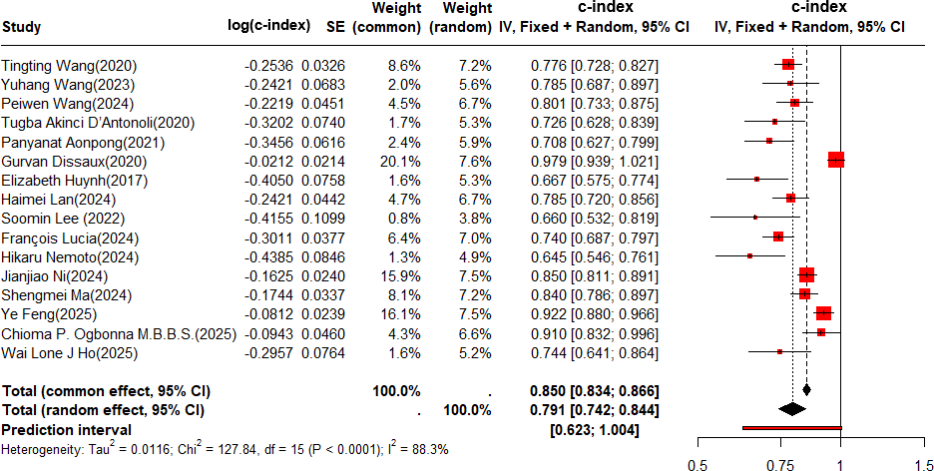
Forest plot for meta-analysis of the c-index of radiomics-based machine learning (ML) for predicting risk of recurrence in non–small cell lung cancer (NSCLC) in the training set. The forest plot was analyzed using a random effects model and corrected by the Knapp-Hartung method [20-35]. DBP: diastolic blood pressure; OR: odds ratio; SBP: systolic blood pressure.

In the training set, the c-index was pooled by REM (τ^2^=0.0116; τ=0.1035; *I*^2^=88.3%), and the c-index of the radiomics-based ML for predicting the risk of recurrence in NSCLC was 0.850 (95% CI 0.834‐0.866, 95% prediction interval [PI] 0.623‐1.000; Figure 2).

The pooled c-index was 0.876 (95% CI 0.853‐0.900) among the patients receiving SBRT, and 0.825 (95% CI 0.804‐0.848) among those who received surgeries combined with other adjuvant treatment regimens (Figure S1 in Multimedia Appendix 1). The pooled c-index was 0.815 (95% CI 0.776‐0.856) among the patients with adenocarcinoma and squamous cell carcinoma as the pathological type, and 0.856 (95% CI 0.839‐0.874) among other patients with unspecified specific pathological types (Figure S2 in Multimedia Appendix 1). The pooled c-index was 0.851 (95% CI 0.832‐0.870) among the patients with early-stage NSCLC, and 0.848 (95% CI 0.818‐0.879) among the patients at a locally advanced stage and an advanced stage (Figure S3 in Multimedia Appendix 1).

**Figure 3.**
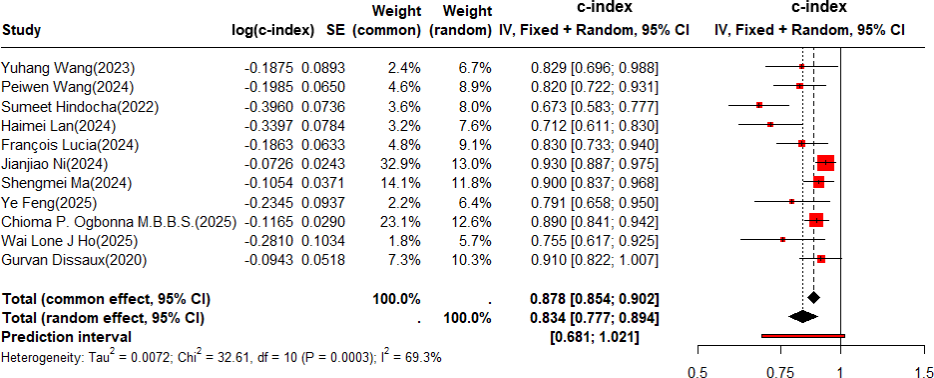
Forest plot for meta-analysis of the c-index of radiomics-based machine learning for predicting risk of recurrence in non–small cell lung cancer in the validation set. The forest plot was analyzed using a random effects model and corrected by the Knapp-Hartung method [21-30,36]. IV: independent variable.

In the validation set, the c-index was pooled by REM (τ^2^=0.0072; τ=0.0750; *I*^2^=69.3%), and the c-index of the radiomics-based ML for predicting the risk of recurrence in NSCLC was 0.878 (95% CI: 0.854‐0.902, 95% PI 0.681‐1.000; Figure 3).

The pooled c-index was 0.907 (95% CI 0.877‐0.937) among the patients receiving SBRT, and 0.819 (95% CI 0.780‐0.859) among those who received surgeries combined with other adjuvant treatment regimens (Figure S4 in Multimedia Appendix 1). The pooled c-index was 0.858 (95% CI 0.807‐0.913) among the patients with adenocarcinoma-squamous cell carcinoma as the pathological type, and 0.883 (95% CI 0.856‐0.910) among other patients with unspecified specific pathological types (Figure S5 in Multimedia Appendix 1). Among the patients with early-stage NSCLC, the pooled c-index was 0.896 (95% CI 0.870‐0.922), and among the patients at a locally advanced stage and an advanced stage, the pooled c-index was 0.759 (95% CI 0.702‐0.821; Figure S6 in Multimedia Appendix 1). In the subgroup with fewer than 100 cases, the pooled c-index was 0.851 (95% CI 0.815‐0.888), while in the subgroup with more than 100 cases, it was 0.897 (95% CI 0.865‐0.929; Figure S7 in Multimedia Appendix 1).

### ML Based on Radiomics Combined With Clinical Features

**Figure 4.**
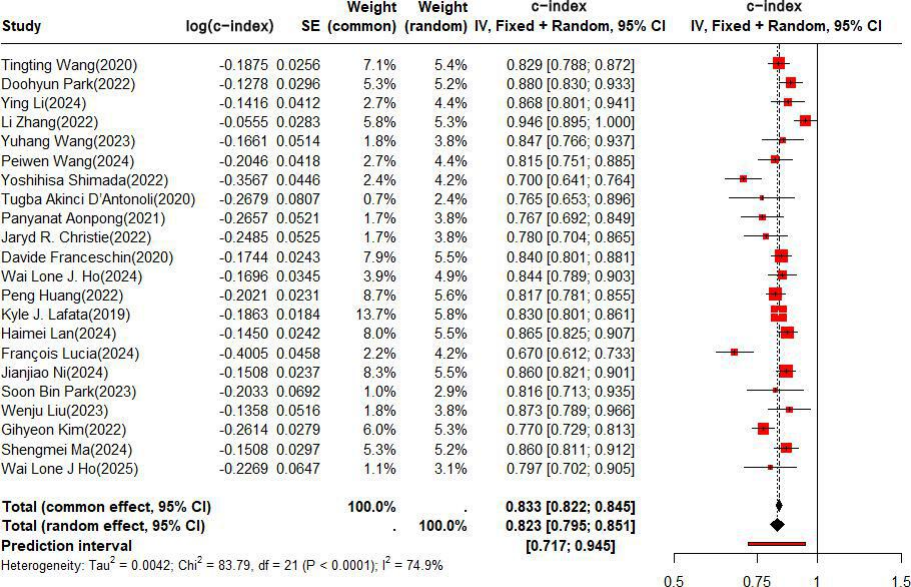
Forest plot for meta-analysis of the c-index of radiomics-based machine learning combined with clinical features for predicting risk of recurrence in non–small cell lung cancer in the training set. The forest plot was analyzed using a random effects model and corrected by the Knapp-Hartung method [20-22,24-27,30-32,37-48]. IV: independent variable.

In the training set, the c-index was pooled by REM (τ^2^=0.0042; τ=0.0628; *I*^2^=74.9%), and the c-index of the ML based on radiomics combined with clinical features for predicting the risk of recurrence in NSCLC was 0.833 (95% CI 0.822‐0.854, 95% PI 0.717‐0.945; Figure 4).

The pooled c-index was 0.828 (95% CI 0.808‐0.848) among the patients receiving SBRT, and 0.836 (95% CI 0.823‐0.850) among the patients who received surgeries combined with other adjuvant treatment regimens (Figure S8 in Multimedia Appendix 1). The pooled c-index was 0.882 (95% CI 0.858‐0.907) among the patients with adenocarcinoma-squamous cell carcinoma, and 0.823 (95% CI 0.810‐0.837) among other patients with unspecified specific pathological types (Figure S9 in Multimedia Appendix 1). Among the NSCLC patients, the pooled c-index was 0.842 (95% CI 0.829‐0.855), and among the patients at a locally advanced stage and an advanced stage, the pooled c-index was 0.802 (95% CI 0.779‐0.826; Figure S10 in Multimedia Appendix 1).

**Figure 5.**
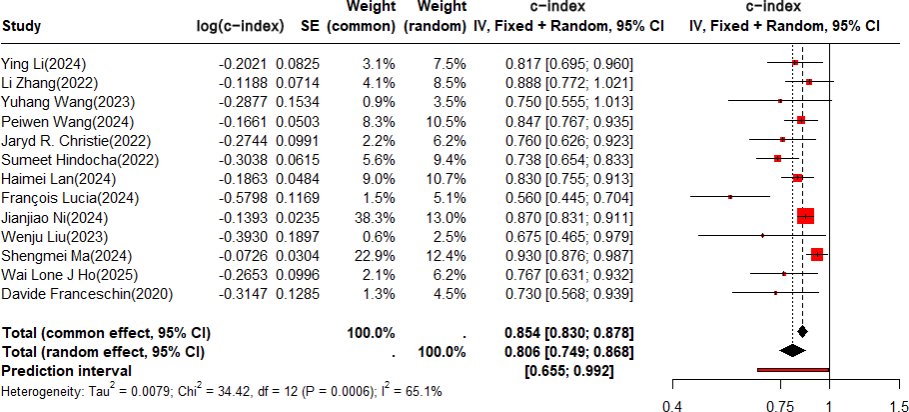
Forest plot for meta-analysis of the c-index of radiomics-based machine learning combined with clinical features for predicting risk of recurrence in non–small cell lung cancer in the validation set. The forest plot was analyzed using a random effects model and corrected by the Knapp-Hartung method [21,22,24-27,30,36,38,39,41,44,46]. IV: independent variable.

In the validation set, the c-index was pooled by REM (τ^2^=0.0079; τ=0.1069; *I*^2^=65.1%), and the c-index of the ML based on radiomics combined with clinical features for predicting the risk of recurrence in NSCLC was 0.854 (95% CI 0.830‐0.878, 95% PI 0.655‐0.992; Figure 5).

The pooled c-index was 0.851 (95% CI 0.814‐0.890) among the patients receiving SBRT, and 0.855 (95% CI 0.8824‐0.888) among the patients who received surgeries combined with other adjuvant treatment regimens (Figure S11 in Multimedia Appendix 1). The pooled c-index was 0.889 (95% CI 0.850‐0.930) among the patients with adenocarcinoma-squamous cell carcinoma, and 0.831 (95% CI 0.801‐0.862) among other patients with unspecified specific pathological types (Figure S12 in Multimedia Appendix 1). Among the patients with early-stage NSCLC, the pooled c-index was 0.870 (95% CI 0.842‐0.897), and among the patients at a locally advanced stage and an advanced stage, the pooled c-index was 0.788 (95% CI 0.738‐0.842; Figure S13 in Multimedia Appendix 1). The pooled c-index in the subgroup with fewer than 100 cases was 0.855 (95% CI 0.823‐0.888), while in the subgroup with more than 100 cases, it was 0.852 (95% CI 0.816‐0.889; Figure S14 in Multimedia Appendix 1).

## Discussion

### Principal Findings

That meta-analysis of 30 cohort studies demonstrated that the radiomics-based ML achieved pooled c-indexes of 0.850 in the training set and 0.878 in the validation set for predicting recurrence in NSCLC. These findings indicate that radiomics, as a noninvasive and reproducible imaging analysis technique, holds clinical potential for predicting the risk of recurrence in NSCLC. Most studies, for the purpose of improving the prediction accuracy and strengthening the interpretability of the radiomics-based ML, tend to combine clinical features with radiomics features. However, this study revealed that although these attempts indeed enhanced the c-index of the ML model, they failed to show remarkable advantages. It is plausible that radiomics features may inherently capture certain clinically relevant information associated with recurrence, while the clinical features added in the above studies vary greatly and fail to fully reflect the biological characteristics of patients. Therefore, the additional clinical features of patients lead to a limited improvement in the predictive efficiency of the model, which, to some extent, reflects that the radiomics-based model may be more stable.

To thoroughly ascertain the predictive efficiency of the radiomics-based model, subgroup analyses were conducted from multiple dimensions such as stage, treatment modality, and pathological classification. Regarding disease stage, the c-index of the model in patients with NSCLC at an early stage was 0.96, which was higher than that in patients with locally advanced NSCLC (c-index=0.759). The tumors in early-stage NSCLC are usually well-defined in terms of boundaries and relatively intact in structure, and the imaging features of the tumors are more easily recognized and quantified by the model, thereby improving the predictive stability and discriminative capacity of the models. In contrast, the tumors in patients with locally advanced NSCLC often invade adjacent areas such as the mediastinum, pericardium, and chest wall, or present lymph node metastases such as at the pulmonary hilum and mediastinum, and some patients may also suffer from complications such as obstructive pneumonia. These factors contribute to more complex imaging features and increased heterogeneity, which may interfere with the extraction and learning of the model for key features and thereby affect the prediction accuracy [49,50]. Additionally, patients with locally advanced NSCLC often undergo treatment interventions, including neoadjuvant chemotherapy and radiotherapy, which may further change the expression patterns of imaging features [51].

The subgroup analyses, particularly those with wide CIs, should be interpreted with caution. This study included 7 studies using SBRT and 18 studies utilizing surgical treatment. Although the c-index of the 2 groups was similar, the CI of the SBRT group was significantly wider, suggesting that the stability of the prediction results in this group was poorer. These findings are exploratory and may reflect variability due to small sample sizes or heterogeneity among included studies. Therefore, the subgroup results should be considered hypothesis-generating rather than conclusive. Future research with larger datasets and consistent analytical frameworks is warranted to confirm these preliminary trends. Moreover, SBRT serves as a relatively new and highly technology-dependent treatment modality, and there are significant differences in implementation plans, dose settings, and patient selection criteria among different studies, which may also lead to increased uncertainty in the performance evaluation of the model [52]. These results imply that the stability of current radiomics-based models in patients undergoing SBRT or other nonsurgical treatments still requires further verification. In the future, more rigorous prospective investigations with larger sample sizes are needed to assess the applicability and reliability of the radiomics-based model in patients with NSCLC receiving SBRT treatment, thereby facilitating its integration into personalized treatment plans.

In terms of pathological classification, a total of 22 studies in this study only mentioned NSCLC without further subdividing pathological subtypes (squamous cell carcinoma and adenocarcinoma). The findings revealed that the radiomics-based prediction model exhibited superior predictive accuracy in studies classifying adenocarcinoma and squamous cell carcinoma than in those without specific subtype classification. Adenocarcinoma and squamous cell carcinoma are the 2 most prevalent pathological subtypes; furthermore, due to their histological heterogeneity, they may be more identifiable in imaging features, thereby enabling the model to extract more discriminative imaging features and improving the prediction accuracy [53,54]. In contrast, in studies that did not explicitly include information on pathological subtypes, the predictive performance of their models may be limited due to high sample heterogeneity and inconsistent imaging features. In training and validating subsequent radiomics-based models, explicit histological classifications should be included as much as possible to strengthen the stability and the practical applicability of models. Moreover, given the distinct biological characteristics, therapeutic responses, and prognoses across subtypes, combining histopathological information for multimodal fusion modeling may enhance the efficacy of the radiomics-based prediction models. In summary, while the subgroup analysis is supported by the meta-analysis and the sensitivity analysis, the findings should be considered preliminary and hypothesis-generating. In the future, additional prospective studies with a large sample size and based on multicenter data are warranted to validate these findings.

Radiomics has achieved considerable progress in predicting the risk of recurrence in NSCLC. However, due to the inherent complexity of radiomics and the limited reproducibility of the numerous processes involved, it still faces many challenges in clinical applications. Particularly, in image segmentation, feature extraction, and dataset analysis, the application of radiomics is still limited to academic research [55-57]. To begin with, a primary technical challenge in current radiomics research is the absence of standardized imaging protocols [58]. Different medical institutions use scanning equipment and image acquisition parameters that vary greatly. Even for the same patient, the imaging quality and image features obtained at different times or with different devices may vary, thereby affecting the stability of feature extraction and the generalization ability of models [55,59-62]. Standardizing the processing of image data across devices and centers may be the key to improving the clinical applicability of models. Currently, some studies attempt to reduce the influence of image differences through image normalization, resampling, and image domain adaptation [59], but more confirmatory research is needed. In the future, pilot studies with small samples may be conducted prior to formal model construction to evaluate the actual impact of image protocol differences on model construction. Given the subjectivity and consistency issue of the image segmentation process, the stability of models is affected, and manual segmentation is influenced by operator expertise, leading to variability [58,63,64]. Therefore, more studies attempt to apply deep learning methods for automatic or semiautomatic segmentation and to promote the application of automatic segmentation algorithms in multidisease and multimodal data to improve efficiency and reproducibility [65-67].

Moreover, radiomics usually involves high-dimensional small-sample data, so it is frequently associated with overfitting, whereby the radiomics-based model performs well on training data but underperforms on new data. This issue may be alleviated through techniques such as feature selection and data augmentation. In the previous study by Ye et al [68], the robustness of the model was improved through data augmentation and feature selection, and kernelization methods were used, and principal component analysis was performed to lower the feature dimension to effectively reduce the risk of overfitting. In summary, as an emerging tool for imaging analysis, radiomics holds potential for clinical practice. Nonetheless, before radiomics is applied in clinical practice, the above-mentioned problems should be addressed.

It should be noted that the overall methodological quality of the included studies, as reflected by the relatively low mean RQS (27.4%), indicates substantial limitations in current radiomics-based ML research. Many of the included studies were absent from external validation, prospective design, or standardized feature extraction procedures, which may introduce bias and restrict the generalizability of the pooled diagnostic accuracy. Therefore, the results of this meta-analysis should be interpreted with caution. Future studies are encouraged to adhere to standardized radiomics workflows and reporting guidelines, and to perform multicenter validations to enhance methodological rigor and clinical reliability. Furthermore, the overall RQS of the included studies was relatively low, primarily manifested in the following aspects: biological relevance, cost-benefit analysis, comparison with the gold standard, and prospective design. Biological relevance involves the link of imaging features with genetic or molecular features; however, only 1 included study reported the potential correlation of gene levels and imaging features. None of the original studies included were prospectively registered, and some were absent from external validation or solely relied on internal validation based on single-center data. These deficiencies collectively led to the low RQS.

### Strengths and Limitations

Although this meta-analysis demonstrates the potential of radiomics-based ML for forecasting the risk of recurrence in NSCLC, certain limitations still need to be resolved to strengthen the reliability and clinical applicability of radiomics-based models for the prediction of the risk of recurrence in NSCLC. First, this study primarily utilizes retrospective data, potentially introducing selection bias and other issues, thereby limiting the generalizability of the research results. Second, although subgroup analyses are conducted, it is still difficult to completely eliminate heterogeneity. Finally, some studies are limited to the sample sizes, lack external validation, and lack long-term follow-up analysis of actual clinical prognosis, which restricts the assessment of the practical applicability of radiomics-based models.

### Conclusion

This systematic review, for the first time, confirms that radiomics-based ML models effectively predict the risk of recurrence in NSCLC, and clinically relevant subgroups (eg, model type, treatment background, stage, and pathology) were explored. Differing from individual original studies or extensive reviews focusing on radiomics with mixed heterogeneous end points, this study concentrates on recurrence, a clinically operable outcome indicator, thereby clarifying areas where model performance is most pronounced and methodological gaps are identified. In clinical practice, robust radiomics-based ML tools may be leveraged to support individualized monitoring intensity, aid in formulating treatment regimens, and optimize risk stratification in clinical trials. Nonetheless, the current application of radiomics methodologies remains insufficient. Therefore, in the future, studies should focus on standardizing the implementation procedure of radiomics-based ML and integrating multicenter imaging data to enhance generalizability.

## Supplementary material

10.2196/84223 Multimedia Appendix 1 Supplemental tables and figures: the detailed literature search strategies (Table S1), characteristics of the included studies (Table S2), Radiomics Quality Score assessments (Table S3), and forest plots depicting subgroup analyses for the meta-analysis of machine learning models predicting recurrence in non–small cell lung cancer (Figures S1-S14).

10.2196/84223 Checklist 1 PRISMA checklist.

10.2196/84223 Checklist 2 PRISMA-S checklist.

## Abbreviations

CT: computed tomography
ML: machine learning
NSCLC: non–small cell lung cancer
PET-CT: positron emission tomography–computed tomography
PRISMA: Preferred Reporting Items for Systematic Reviews and Meta-Analyses
PRISMA-S: Preferred Reporting Items for Systematic Reviews and Meta-Analyses–Search Extension
REM: random effects model
RQS: Radiomics Quality Score
SBRT: stereotactic body radiation therapy
TNM: tumor node metastasis
